# Integrated LiDAR-Based Localization Correction Using a Dedicated Support Sign for Autonomous Vehicles

**DOI:** 10.3390/s26123941

**Published:** 2026-06-21

**Authors:** Yuseung Oh, Seungyeon Jang, Ilseung Yoon, Bumjin Park, Byeongsup Moon

**Affiliations:** 1 Department of Highway and Transportation Research, Korea Institute of Civil Engineering and Building Technology, Goyang-si 10223, Republic of Korea; mr.ys.oh@gmail.com (Y.O.); jseunggle401@kict.re.kr (S.J.); park_bumjin@kict.re.kr (B.P.); 2 RideFlux Inc., Jeju-si 63088, Republic of Korea; y1seung@rideflux.com

**Keywords:** autonomous vehicle, LiDAR, localization correction, point cloud registration, GNSS-degraded tunnel section, dedicated support sign

## Abstract

**Highlights:**

**What are the main findings?**

- A dedicated LiDAR support sign was integrated into a localization pipeline.
- The proposed pipeline reduced localization drift relative to GNSS/INS-only localization and correction based on ordinary tunnel structures.

**What are the implications of the main findings?**

- Dedicated signs provide more stable references in GNSS-degraded tunnel sections.
- LiDAR-based correction can support localization in GNSS-degraded tunnel sections.

**Abstract:**

Accurate vehicle localization must be maintained even in tunnel sections where GNSS reliability is degraded. However, conventional GNSS/INS-based localization rapidly accumulates errors in such environments, affecting lane-level decision-making and path-following stability. To address this problem, this study proposes a dedicated localization support sign for stable LiDAR observation and a point-cloud-registration-based correction algorithm. The proposed method detects a dedicated sign using a PointPillars-based detector, and the corresponding point cloud is registered to a pre-built reference map to estimate a rigid correction transform online. The sign was installed in a tunnel section of a proving ground that reproduces real-road conditions. For evaluation, the driving sequence was analyzed by separating the pre-entry section, the tunnel section before dedicated-sign recognition, and the section after dedicated-sign recognition. The proposed pipeline substantially reduced localization error after dedicated-sign recognition, compared with the GNSS/INS-only baseline. The dedicated sign also provided more stable correction than ordinary tunnel structures within the same registration pipeline. These results indicate that the proposed LiDAR-based pipeline can suppress localization drift in GNSS-degraded sections.

## 1. Introduction

Autonomous vehicles must reliably estimate their position and pose throughout the perception, decision-making, planning, and control processes. In Level 3+ automated driving, lane-level accuracy is required. Therefore, positioning errors affect not only the coordinates but also lane assignment, path tracking, lane-change timing, and the consistency of high-definition map matching. In tunnels, underground roads, urban canyons, work zones, and dense high-rise corridors, reduced GNSS reliability and accumulated INS errors can jointly accelerate localization drift. This makes localization a major bottleneck in autonomous driving systems [1,2].

Vehicle localization is typically implemented using a combined GNSS/INS architecture and is often supplemented by proprioceptive vehicle sensors, such as wheel-speed and steering-angle sensors, as well as high-definition map matching with LiDAR, radar, or cameras. However, GNSS is strongly affected by reduced satellite visibility, multipath, and structural occlusions. INS is stable in the short term; however, its error accumulates over time. As a result, GNSS/INS alone cannot maintain a stable localization quality over long occluded sections, and correction using external reference structures is necessary [3,4,5].

Combinations of cameras, radar, LiDAR, and GNSS have been widely studied because each sensor can compensate, to some extent, for the weaknesses of the others under normal conditions [2,3,6,7,8,9,10]. Under degraded driving conditions, however, one or more supporting sensors may no longer perform at their nominal level. Weather changes, low illumination, fog, rain, and structural occlusions can all reduce the reliability of auxiliary sensors and make system-level decisions more difficult. This study does not experimentally reproduce all such degraded conditions; rather, it focuses on a tunnel section where GNSS availability is structurally limited. For this reason, the present study focuses on localization support based on LiDAR alone, without relying on auxiliary sensors. LiDAR was selected as the reference sensor because it can provide relatively robust 3D geometric information under such conditions. A dedicated localization support sign was therefore designed to provide a LiDAR-observable geometric reference in GNSS-degraded tunnel sections [11,12,13].

Recently, many studies have explored LiDAR-based localization using high-definition maps, road infrastructure, scan matching, SLAM, and sparse landmark references. These approaches reduce localization errors by registering current observations to absolute coordinates on a map [6,10,14,15,16,17,18,19,20,21,22,23,24]. However, their practical use on public roads faces two major constraints. First, they require detailed maps and close integration with those maps, while robust operation often depends on near-real-time map maintenance. Second, ordinary road structures are difficult to use as repeatable landmark references because their surface reflectance, geometry, installation condition, damage state, and maintenance quality change over time.

The main distinction of this study lies in the design of a dedicated localization support sign that gives practical meaning to the correction algorithm. The proposed correction pipeline was built around a sign that can be recognized repeatedly by LiDAR without being confused with ordinary roadside structures. The workflow was then configured so that the sign could be detected, registered, and used for localization correction within one end-to-end process. This study is also meaningful because that combined workflow was quantitatively verified in a proving-ground tunnel that reproduces real-road conditions.

The contributions of this study are as follows. First, this study proposes an infrastructure-assisted localization-correction framework in which a dedicated LiDAR-observable sign functions as a repeatable geometric anchor in GNSS-degraded tunnel sections. Second, the study integrates sign detection, predefined support-point use, source–target point-cloud registration, and pose correction into a single end-to-end workflow, thereby linking physical infrastructure design with online localization correction. Third, the validation protocol separates GNSS/INS-only localization, correction based on ordinary tunnel structures, and correction based on the dedicated support sign, allowing the contribution of the dedicated infrastructure to be evaluated independently. Fourth, the study provides empirical evidence that a purpose-built LiDAR localization sign can provide a more stable correction reference than ordinary tunnel structures within the same registration pipeline.

The remainder of this paper is organized as follows. Section 2 reviews prior studies relevant to localization in GNSS-degraded environments. Section 3 describes the experimental environment, vehicle, and data acquisition procedure. Section 4 presents the methodology and validation protocol, including sign detection and point cloud registration. Section 5 reports the results of the analysis. Section 6 discusses the findings, limitations, and practical implications, and Section 7 concludes the paper. The key terms used in this study are summarized in Appendix A.

## 2. Literature Review

### 2.1. GNSS/INS-Based Localization and Challenges in Autonomous Driving

The traditional basis of autonomous vehicle localization is a combination of GNSS and INS. GNSS provides an absolute coordinate reference, and INS provides continuous and short-term estimates of motion and pose. However, in real road environments, GNSS quality can degrade rapidly because of reduced satellite visibility, multipath, structural occlusion, and signal shadowing. In such cases, INS-based dead reckoning compensates for the missing information; however, the error accumulates over time and often appears as an increasing longitudinal drift. For lane-level autonomous driving, this accumulated error becomes a direct safety-related problem [2,3,4,25,26].

Wheel-speed and steering-angle sensors are also widely used as proprioceptive vehicle sensors for localization and dead reckoning. When fused with INS and GNSS, these measurements can constrain traveled distance and yaw motion, thereby reducing short-term drift in GNSS-limited environments [27,28]. However, vehicle-odometry-based constraints remain sensitive to wheel slip, tire–road interaction, calibration errors, and accumulated integration errors; therefore, they are complementary to external correction references rather than a complete substitute for absolute or map-based localization updates [28,29].

Previous studies have treated this issue not as a simple sensor noise problem but as a core technical challenge in degraded autonomous driving conditions. Accurate vehicle localization is a prerequisite for perception, decision-making, and control in tunnels, work zones, dense high-rise districts, and severe weather. Therefore, localization remains one of the first conditions that must be secured when degraded automated driving is considered [5,12,13].

### 2.2. LiDAR-Inertial Odometry in GNSS-Degraded Environments

LiDAR-inertial odometry (LIO) has become an important approach for localization in GNSS-degraded or GNSS-denied environments because it combines the geometric information obtained from LiDAR scans with the short-term motion constraints provided by inertial measurements. In general, LiDAR observations are used to correct geometric alignment, while IMU measurements support motion prediction, scan de-skewing, and continuous pose estimation between LiDAR frames. Therefore, LIO methods can reduce the drift that occurs when GNSS updates are unavailable and have been widely adopted as real-time odometry and mapping frameworks.

Several representative LIO frameworks have been proposed in recent years. LIO-SAM formulates tightly coupled LiDAR-inertial odometry as a factor-graph-based smoothing and mapping problem, allowing relative and absolute measurements, including loop closures, to be incorporated as factors [30]. FAST-LIO2 improves computational efficiency and robustness by using a tightly coupled iterated Kalman filter and directly registering raw points to the map without explicit feature extraction [31]. DLIO further emphasizes lightweight computation and continuous-time motion correction by constructing continuous-time trajectories for precise point-wise deskewing [32]. More recent frameworks, such as GLIM, introduce GPU-accelerated scan-matching factors and fixed-lag smoothing to improve robustness and reduce drift even under partially degenerated range observations [33]. MOLA also provides a modular localization and mapping framework that supports configurable LiDAR odometry, map manipulation, and localization pipelines [23].

Despite these advances, LIO methods can still be affected by the geometric characteristics of the surrounding environment. In tunnel sections, the scene often consists of repetitive walls, limited geometric variation, and elongated corridor-like structures. These conditions can weaken scan-matching constraints and make localization more vulnerable to geometric degeneracy and accumulated drift. Therefore, the present study does not attempt to replace LIO-based localization frameworks. Instead, it focuses on a complementary infrastructure-assisted correction strategy in which a dedicated LiDAR-observable support sign provides a repeatable geometric reference for drift correction in GNSS-degraded tunnel sections.

### 2.3. LiDAR and Map-Based Localization

To address these limitations, LiDAR-based HD map localization and scan matching have been studied extensively. Various map representations have been proposed, including dense intensity, point cloud, surfel, and semantic maps. Recently, sparse semantic maps have been explored to reduce maintenance costs [15,20,21,24,25]. Related studies have also examined semantic generalized ICP, LiDAR descriptor-based global localization, and multi-session map localization [16,17,18,22,34,35].

Dense map-based approaches also have clear constraints. They are expensive to build and update, and they remain sensitive to environmental changes, which means that high-precision maps must be maintained continuously. In addition, general scan matching requires a sufficiently structured scene. When the initial alignment is poor or the correspondence quality is low, the optimization may converge to a local minimum [10,19,23,36]. Moreover, even when many mapped structures are present inside a tunnel, they do not necessarily provide comparable registration quality. Simply having multiple structures in the scene does not guarantee stable correction.

### 2.4. General Landmark-Based Localization

Landmark-based localization reduces the computational and map-building costs using a limited number of reference structures. Previous studies have considered poles, road-marking corners, traffic signs, lane markings, and artificial markers as potential landmarks. However, the key issue is not simply whether a structure exists or not. The question is whether this structure can be used as a repeatable geometric reference. Installation height, orientation, surface damage, repainting, partial occlusion, geometric similarity, and maintenance variations can degrade landmark quality [12].

Some studies have used visual artificial markers for this purpose. These markers require a decodable visual code and are relatively vulnerable to nighttime conditions, fog, rain, contamination, and backlighting [3,7,13]. In contrast, LiDAR is less directly affected by illumination changes and can use 3D geometry directly [1,9,11]. Therefore, a dedicated structure designed for reliable LiDAR observation can provide a more consistent landmark reference than ordinary roadside structures.

The dedicated localization support sign used in this study was not introduced merely to improve detection performance. It was introduced to address two issues simultaneously: a sensor strategy for degraded conditions and a consistent landmark definition. First, degraded driving conditions can include cases in which not all sensors in a multi-sensor suite remain fully reliable. Using LiDAR as the reference sensor is therefore reasonable because it remains comparatively robust to illumination changes and can exploit 3D geometry directly. Second, ordinary roadside structures rarely provide an unambiguous reference point. The dedicated localization support sign was designed so that its support point could be defined in advance and observed repeatedly with LiDAR. Accordingly, this study differs from previous landmark-localization studies in two ways. First, it uses a dedicated localization support sign designed specifically for LiDAR. Second, it directly connects the detection result of that sign to a source–target point-cloud registration pipeline. This study focuses on the quantitative effect produced when the dedicated sign and the correction algorithm operate as one correction workflow.

## 3. Experimental Environment and Data Acquisition

### 3.1. Experimental Environment

**Figure 1:**
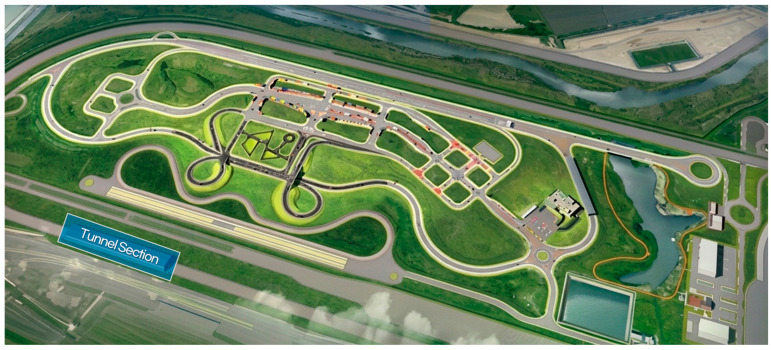
Overview of the K-City test environment and tunnel section.

The performance of the proposed method was validated on 10 November 2025 in the weather simulation tunnel of K-City, which is a nationally certified autonomous-driving test site that reproduces real-road conditions (Figure 1). The test route started from a fixed point on the approach road outside the tunnel and passed through a dedicated localization support sign installed inside the tunnel. After each run, the logged trajectory was time-synchronized and compared with the reference trajectory. The sign was installed near the central part of the tunnel. Therefore, the vehicle experienced correction based on general tunnel structures in the earlier part of the tunnel and correction based on the dedicated localization support sign in the later part.

### 3.2. Autonomous Vehicle and Sensor Specifications

**Figure 2:**
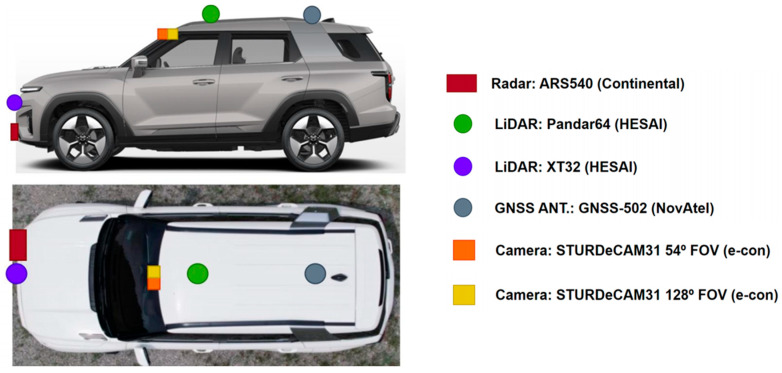
Experimental vehicle and sensor mounting configuration.

The test vehicle was a five-passenger autonomous vehicle based on an electric passenger car platform. As shown in Figure 2, it is equipped with LiDAR, radar, GNSS, and cameras. The reference sensor for sign recognition and localization correction in this study was a rooftop LiDAR. The vehicle platform completed the Korean temporary permit test for autonomous driving in October 2024. Preliminary verification of the proposed recognition and correction algorithm was conducted in October 2025.

**Figure 3:**
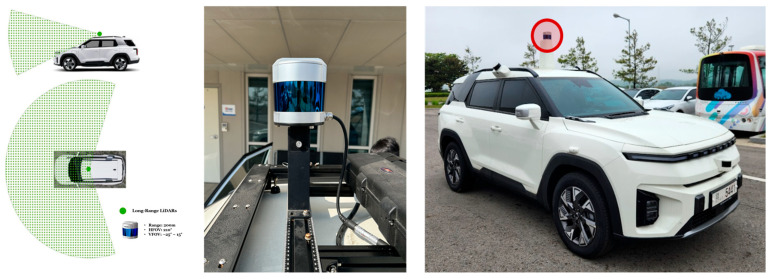
Specification of the Pandar64 LiDAR sensor used for sign recognition and localization correction, vehicle mounting example.

The primary LiDAR used for sign recognition and effectiveness evaluation was the Hesai Pandar64 (Hesai Technology Co., Ltd., Shanghai, China), a 64-channel rotating LiDAR (Figure 3). Its main specifications are as follows: horizontal angular resolution of 0.2–0.4°, maximum vertical angular resolution of 0.167°, vertical field of view of 40° (−25° to +15°), horizontal field of view of 360°, maximum detection range of 200 m at 10% reflectivity, and frame rate of 10 or 20 Hz. The point rate was approximately 1,152,000 points/s in the single-return mode and 2,304,000 points/s in the dual-return mode.

### 3.3. LiDAR-Specific Localization Support Sign

**Figure 4:**
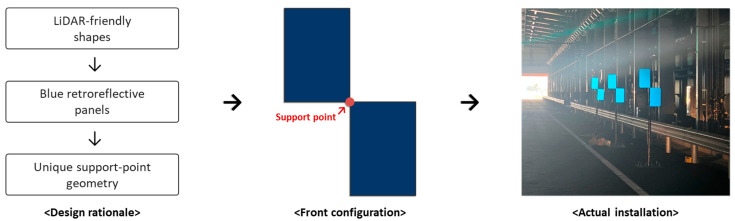
Facility geometry and positioning support-point concept.

The dedicated localization support sign used in this study was a LiDAR-specific reference structure proposed by Kim et al. [12]. The need for such a dedicated sign arises from a practical limitation of ordinary roadside structures: even when they are visible, they do not always provide a unique and repeatable geometric point for localization because their installation height, local geometry, maintenance condition, and surrounding clutter vary across sites. As shown in Figure 4, the sign has a four-quadrant rectangular front. Two quadrants were covered with blue retroreflective sheeting, and the other two were left open. This configuration was selected to satisfy both detectability and geometric distinctiveness. LiDAR observes planar rectangular geometry and edges relatively stably, whereas retroreflective material provides a strong and consistent return intensity [11].

The sign was 800 mm high and 460 mm wide and was installed at a height of 1300 mm. The reference point used for localization correction was the corner formed by the intersection of two rectangular planes. This point is defined as the localization-support point. It is not a generic visual center; rather, it is the predefined geometric point used to connect the detected structure to the absolute coordinates stored in the map. The cross-origin configuration was adopted so that one repeatable support point could be estimated from LiDAR point clouds even when the visible points vary slightly with distance and viewpoint. This directly addresses a common weakness of ordinary road signs and tunnel structures, for which the choice of a reference point can remain ambiguous in a 3D coordinate frame. Once the support point is estimated, the observed sign cloud can be matched one-to-one with the mapped sign cloud, and the resulting rigid transform can be used to realign the vehicle pose to the absolute frame. In this sense, the dedicated sign is meaningful not merely as a detectable object but as sparse localization infrastructure that couples recognition, reference-point definition, and registration stability within the same correction workflow [11].

### 3.4. Data Acquisition Procedure

**Figure 5:**
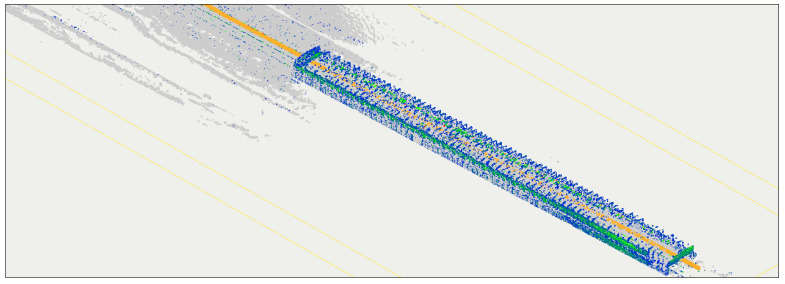
Tunnel section on reproduction simulator for post-processing coordinate reference.

An effectiveness verification experiment was conducted by repeatedly driving along the same route. The repeated route started from a fixed point 125 m before entering the tunnel. The vehicle departed from rest with an active ADS. It then recognized the dedicated localization support sign installed approximately 100 m inside the tunnel and maintained a stable correction result for 5 s after the recognition. The starting point was fixed, and the vehicle followed the K-City tunnel recommended speed for autonomous driving (30 km/h) in autonomous mode. This controlled the timing and trajectory. This speed was selected to comply with the proving-ground safety protocol and to ensure repeatable autonomous-driving trajectories. Therefore, the experiment should be interpreted as a controlled proving-ground validation rather than a full highway-speed validation. After the runs, the logs from the 10 repeated drives were averaged. The corrected and GNSS/INS-only positions were then compared with the absolute reference coordinates derived from a dedicated map. The replay simulation environment implemented for post-processing and validation is illustrated in Figure 5.

The validation log consisted of four synchronized data streams: the post-processed reference trajectory, the GNSS/INS-only baseline, the corrected ego-localization output, and the LiDAR-feature output. These data streams correspond to gt, novatel_debug, ego_debug, and lidar_feature_debug in the log, respectively. The reference-trajectory and LiDAR-feature streams were recorded at an average rate of 9.94 Hz, which is consistent with nominal 10 Hz LiDAR operation, while the GNSS/INS baseline and corrected ego-state streams were recorded at approximately 99.4 Hz.

## 4. Methodology

### 4.1. Localization Correction Pipeline

The problem addressed in this study is defined as follows: Let $P_{k}$ denote the raw LiDAR point cloud acquired at time k, and let $X_{k}^{raw}$ denote the raw vehicle pose. When the dedicated localization support sign is observed in the current frame, the observed sign point cloud $S_{k}$ is registered to the reference sign point cloud M stored in the map. This registration yields the rigid correction transform $\Delta T_{k}$. The corrected pose $X_{k}^{corr}$ is then obtained by applying $\Delta T_{k}$ to $X_{k}^{raw}$.

(1) $$X_{k}^{corr}=\Delta T_{k}X_{k}^{raw}$$

**Figure 6:**
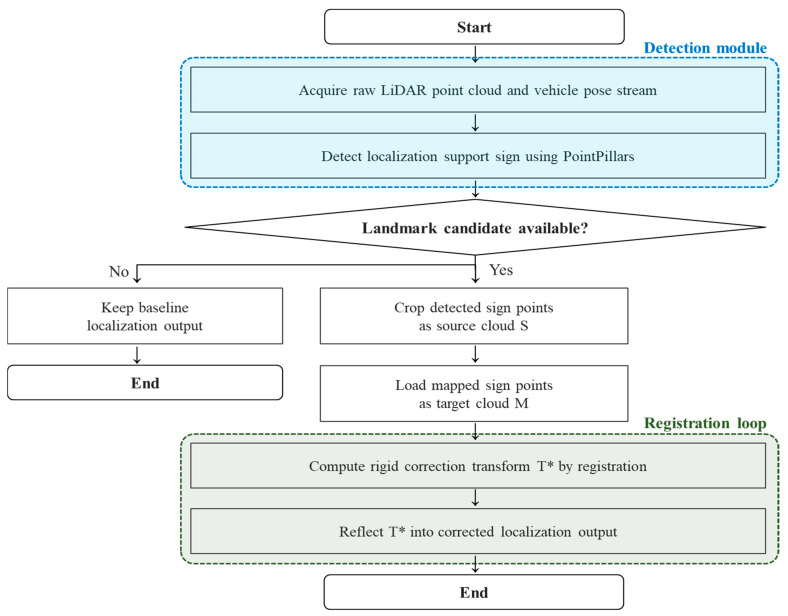
Overall localization-correction pipeline. The asterisk denotes the estimated correction transform obtained from registration.

The pipeline (Figure 6) consists of three stages: First, the dedicated localization support sign is detected and a source cloud is generated for registration. Second, rigid registration is performed between the detected sign point cloud and mapped reference point cloud. Third, the estimated correction transform is applied to the vehicle pose to obtain the corrected position of the vehicle. This study describes the pipeline mainly for the case in which a dedicated localization support sign triggers the correction branch. However, the registration core itself uses the same framework whenever the source and target clouds are available. Therefore, the same registration framework can also operate when other tunnel structures are used as landmark candidates. In this study, the registration module is not intended as a new generic point-cloud registration algorithm; rather, it is used as part of a sign-specific correction workflow in which the predefined support point, controlled sign geometry, and mapped reference cloud constrain the localization-correction problem.

#### 4.1.1. PointPillars-Based Sign Detection Module

The recognition module uses a PointPillars-based 3D object detector [37]. The input point cloud is represented as a four-dimensional point set composed of x, y, z and intensity. First, it is quantized into a fixed-resolution grid on the horizontal plane during pillarization. In this implementation, the voxel size was 0.16 m × 0.16 m × 4.0 m, and the point-cloud range was set to [−8.0, −48.0, −3.0, 0.0, 0.0, 1.0]. Each voxel stored up to 32 points, and the maximum number of voxels per frame was limited to 5000.

For each pillar, the feature vector of the i-th point includes the original coordinates and intensity, the coordinates relative to the mean point within the pillar, and the coordinates relative to the pillar center.

(2) $$f_{i}=\left[x_{i},y_{i},z_{i},I_{i},x_{i}-x_{mean},y_{i}-y_{mean},z_{i}-z_{mean},x_{i}-x_{c},y_{i}-y_{c},z_{i}-z_{c}\right]$$

**Figure 7:**
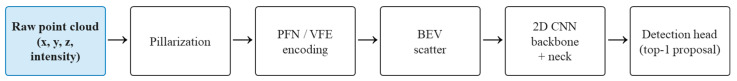
PointPillars-based detection module used for localization-facility recognition.

Here, $x_{mean}$, $y_{mean}$, and $z_{mean}$ are the average coordinates of the points contained in the pillar, and $x_{c}$, $y_{c}$, and $z_{c}$ are the coordinates of the pillar center. These features are compressed into a pillar-level feature through the Pillar Feature Net (PFN) layer using linear transformation, batch normalization, ReLU, and max pooling. The features are then scattered onto a Bird’s-Eye View (BEV) pseudo-image. Multiscale features were generated using a 2D CNN backbone and neck. The overall structure of the detection module is shown in Figure 7.

In this implementation, the backbone channel sizes were 32, 64, and 128, with three, five, and five layers, respectively. The neck used upsampling strides of one, two, and four, and its output channels were 64, 64, and 64. These backbone channels are unrelated to the physical number of LiDAR channels. Instead, they refer to the widths of the feature maps within the 2D CNN that processes the BEV representation. The detection head computes classification and box regression through separate 1 × 1 convolutions. During inference, only the highest-scoring candidate is passed to the next stage. For each candidate, the classification head outputs a logit s, and the final score is obtained through the sigmoid function, as shown below:

The detector configuration used a voxel size of 0.16 m x 0.16 m x 4.0 m, a point-cloud range of [−8.0, −48.0, −3.0, 0.0, 0.0, 1.0], a maximum of 32 points per voxel, a maximum of 5000 voxels, and a maximum static input size of 200,000 points. These settings were selected to keep the sign-recognition module compatible with real-time LiDAR-frame processing while limiting the search space to the region relevant to the tunnel-side localization sign.

(3) $$Score=\frac{1}{1+\exp\left(-s\right)}$$

#### 4.1.2. Dedicated Point-Cloud Registration-Based Position Correction

**Figure 8:**
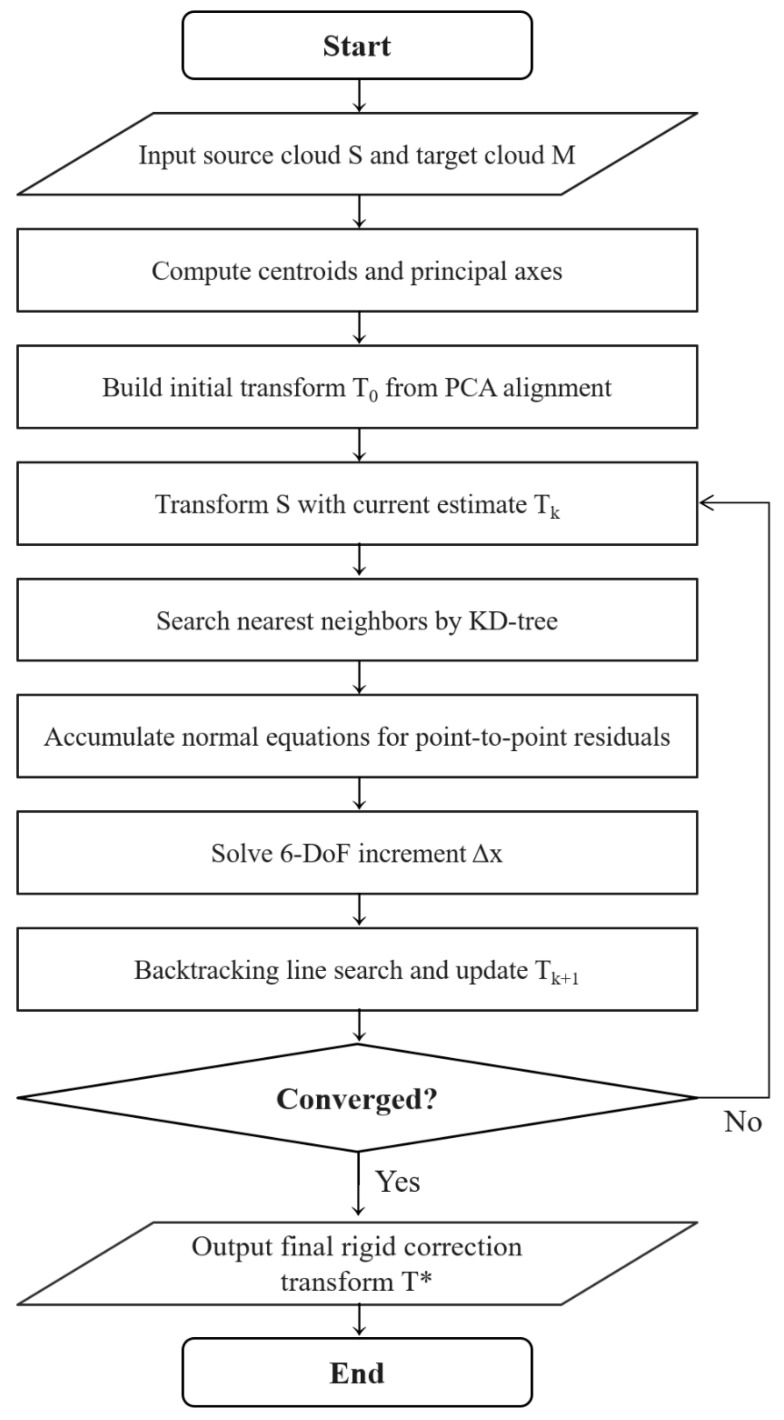
Overall registration loop. The asterisk denotes the final rigid correction transform obtained upon convergence.

Once a sign candidate is detected, the point cloud inside the corresponding bounding box is used as source cloud S. The corresponding mapped sign point cloud is used as the target cloud M. The goal of the registration module is to estimate the rigid transform T that aligns the source cloud with the target cloud. Instead of calling a black-box ICP routine, the implementation uses a PCA-based initial alignment and a custom six-degree-of-freedom linear least-squares registration loop. The overall loop is shown in Figure 8.

**Figure 9:**
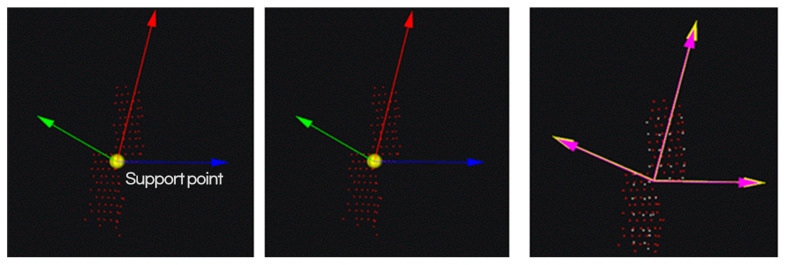
Illustration of alignment based on PCA principal components. Colored arrows indicate the principal component axes used for alignment.

1. PCA-based Initial Alignment
  Let the source cloud be $S_{k}$ and the target cloud be M. The mean of each point cloud $\bar{p}$, $\bar{q}$ is defined as follows:
  (4) $$S_{k}=\{p_{i}{\}}_{\left(i=1\right)}^{N}$$
  (5) $$M=\{q_{j}{\}}_{\left(j=1\right)}^{M}$$
  (6) $$\bar{p}=\frac{1}{N}\sum_{i=1}^{N}p_{i}$$
  (7) $$\bar{q}=\frac{1}{M}\sum_{j=1}^{M}q_{j}$$
  Perform PCA on each point cloud to calculate the principal axis direction matrices $U_{S}$ and $U_{M}$. Using these, define the initial rotation matrix $R_{0}$ and the initial translation vector $t_{0}$ as follows:
  (8) $$R_{0}=U_{M}U_{S}^{T}$$
  (9) $$t_{0}=\bar{q}-R_{0}\bar{p}$$
  Therefore, the initial alignment transform $T_{0}$ is constructed as follows:
  (10) $$T_{0}=\left[\begin{array}{cc} R_{0} & t_{0}\\ 0 & 1 \end{array}\right]$$
  This step offers a rough alignment that helps stabilize the initial values in the iterative registration process. In the implementation, the sign of the last axis is inverted when det($R_{0}$) < 0 to prevent reflection.
  PCA is used only as a coarse initialization step, not as the final registration solution. The final correction transform is estimated through the subsequent KD-tree correspondence search and six-degree-of-freedom least-squares optimization loop. As an auxiliary check, the available reference sign map and a representative detected sign crop showed second-to-third eigenvalue ratios of 9.15 and 122.97, respectively, indicating that the normal direction was distinguishable in the analyzed samples. Nevertheless, if the observed sign point cloud becomes extremely sparse, occluded, or contaminated, PCA-based initialization may still become less reliable; this issue is therefore treated as a limitation and a target for future robust initialization, see Figure 9.
2. Search for Closest Corresponding Points
  After the initial alignment is applied to the source cloud, a KD-tree is built from the target cloud, and the nearest target point $q_{c(i)}$ is searched for each source point $p_{i}$.
  (11) $$c\left(i\right)=\arg\underset{j}{\min}{\left\|q_{j}-p_{i}\right\|}_{2}$$
  That is, the correspondence set is defined as follows:
  (12) $$C=\{(p_{i},q_{c(i)})|i=1,\ldots ,N\}$$
  In the current implementation, each source point is directly assigned to its nearest target point without any separate semantic rejection or distance gating. The reliability of the registration is heavily influenced by the quality of the initial alignment and the geometric consistency of the specific localization support sign.
3. Point-to-Point Least-Squares Registration
  The registration procedure follows a standard ICP framework, but the optimizer itself is implemented as a custom six-degree-of-freedom least-squares solver for point-to-point residuals. Let the currently estimated rigid transform be (R,t). The residual for each correspondence is then defined as follows:
  (13) $$r_{i}=q_{c\left(i\right)}-\left(Rp_{i}+t\right)$$
  The total cost function is defined as follows:
  (14) $$E\left(R,t\right)=\sum_{i=1}^{N}{\left\|r_{i}\right\|}_{2}^{2}$$
  Instead of solving the problem through direct nonlinear optimization, the implementation linearizes it around the current estimate under a small-angle assumption. The corresponding state increment vector is defined as follows:
  (15) $$\Delta x={\left[\Delta t_{x},\Delta t_{y},\Delta t_{z},\Delta \theta_{x},\Delta \theta_{y},\Delta \theta_{z}\right]}^{T}$$
  In the linearized form, each correspondence contributes to the normal equations, whose summation yields the following least-squares problem.
  (16) $$\left(A^{T}A\right)\Delta x=A^{T}b$$
  Here, A is obtained by accumulating the Jacobians of the correspondences, and b is the residual-based right-hand-side vector. In the implementation, each correspondence is visited in sequence, $A^{T}A$ and $A^{T}b$ are accumulated directly, and the resulting linear system is solved to obtain Δx.
4. Transform Update and Line Search
  The calculated increment Δx is not applied at full scale immediately. Instead, the step size is adjusted by backtracking line search before the transform is updated. Let α denote the step size. The actually applied increment is αΔx.
  (17) $$\Delta x^{\prime }=\alpha \Delta x,\;0<\alpha \leq 1$$
  This is converted into a rigid transform ΔT and accumulated in the current transform using left multiplication.
  (18) $$T_{k+1}=\Delta T_{k}T_{k}$$
  In the implementation, the initial step size is 1.0. If the objective function does not decrease, the step size is halved. This process continues until sufficient reduction is achieved or the minimum step-size threshold is reached.
5. Convergence Criteria
  The iteration terminates when two conditions are satisfied simultaneously. First, the norm of the translation increment must be 0.001 m or less. Second, the rotation increment must be 0.25 deg or less. In the implementation, the maximum number of iterations is limited to 20.
  (19) $${\left\|\Delta t\right\|}_{2}<\varepsilon_{t}$$
  (20) $$\left\|\Delta \theta \right\|<\varepsilon_{r}$$
  Upon convergence, the correction transform $\Delta T_{k}$ is output. The final transform is then applied to the raw vehicle pose to obtain the corrected pose $X_{k}^{corr}$.

#### 4.1.3. Implementation Configuration and Computational Feasibility

The PointPillars recognition module was implemented in PyTorch 2.4.1+cu121 and exported to ONNX 1.17.0/ONNX Runtime 1.19.2 and TensorRT 8.5.3 (NVIDIA Corporation, Santa Clara, CA, USA) for deployment-oriented inference. The development environment used for the detector package was Ubuntu 20.04.6 LTS (Canonical Ltd., London, UK), Intel Core i7-12700 CPU (Intel Corporation, Santa Clara, CA, USA), NVIDIA GeForce RTX 3070 GPU (NVIDIA Corporation, Santa Clara, CA, USA), Python 3.8.20 (Python Software Foundation, Wilmington, DE, USA), CUDA 12.8 (NVIDIA Corporation, Santa Clara, CA, USA), and TensorRT 8.5.3.

The implementation package includes a TensorRT timing script that separates inference-only latency, measured using CUDA events, from end-to-end host-device processing latency, measured using a host-side high-resolution timer. The script uses 50 warm-up iterations and 1000 measurement iterations. The recorded LiDAR feature/correction stream operated at approximately 9.94 Hz, which corresponds to a nominal frame budget of about 100 ms per LiDAR frame. Therefore, the implemented recognition and correction pipeline was configured to operate at the 10 Hz LiDAR observed in the field test. Exact deployment latency and peak GPU-memory usage should be re-measured on the final target hardware because they depend on GPU architecture, TensorRT engine build options, host-device transfer configuration, and middleware overhead.

### 4.2. Validation Protocol

**Figure 10:**
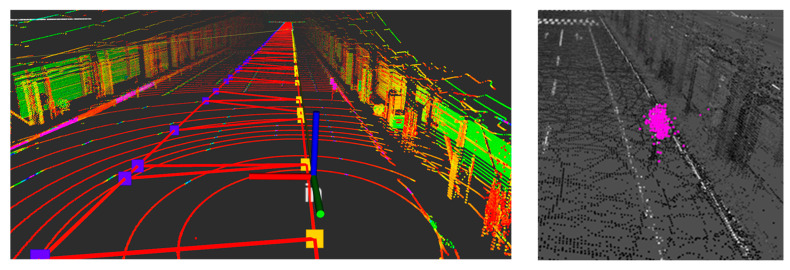
Dedicated map for post-processing the absolute reference coordinates.

The validation of localization-correction performance was performed using time-series position errors extracted from vehicle driving logs. The original logs contain the GNSS/INS-only vehicle position, the final vehicle position after correction, and the position error with respect to the absolute reference coordinates post-processed from the dedicated map, as shown in Figure 10. Only the variables required for position-error analysis were extracted in this study. The x-, y-, and *z*-axis errors were reconstructed, and the overall position error was computed from the magnitude of the three-dimensional error vector. The position error was evaluated with respect to the rear-wheel axle of the vehicle.

The GNSS/INS-only baseline corresponds to the novatel_debug stream, whereas the corrected ego-localization output is represented by the ego_debug stream. For the sake of clarity, rather than relying on a separate numerical GNSS quality index, the GNSS-degraded condition was identified based on the controlled, communication-shadowed tunnel environment and the observed growth in the GNSS/INS-only position error upon tunnel entry. In the latter section of the tunnel, the increasing baseline error indicates that inertial propagation becomes dominant when external GNSS updates are restricted.

The reference trajectory and reference sign map were generated through post-processing using the same experimental reference frame. Accordingly, the reported position errors should be interpreted as relative errors with respect to this post-processed reference trajectory/map, not as an independent claim of centimeter-level absolute map accuracy. This distinction is important when interpreting the directional errors and the overall mean error in the subsequent dedicated-sign correction interval.

As shown in Table 1, the validation was divided into three time intervals. The 0–15 s interval corresponds to the pre-entry section before tunnel entry. The 15–25 s interval corresponds to the section after tunnel entry and before recognition of the dedicated localization support sign. In this interval, correction based on other tunnel structures was active. The 25–30 s interval corresponds to the section after recognition of the dedicated localization support sign. This interval division allows the proposed method to be compared not only with GNSS/INS only but also with correction based on other tunnel structures.

The 25 s boundary should be interpreted as the beginning of the transition toward dedicated-sign-based correction. In the logged sequence, this transition occurs over approximately 25–26 s because recognition, point-cloud cropping, registration, and correction fusion are reflected sequentially in the final ego-localization output.

The evaluation metric is the average position error. The average error along the x, y, and z axes is calculated from the average absolute error at each time point. The overall position error is calculated by averaging the magnitudes of the three-dimensional error vectors at each time point.

## 5. Analysis Results

### 5.1. Analysis Results for the Entire Section

**Figure 11:**
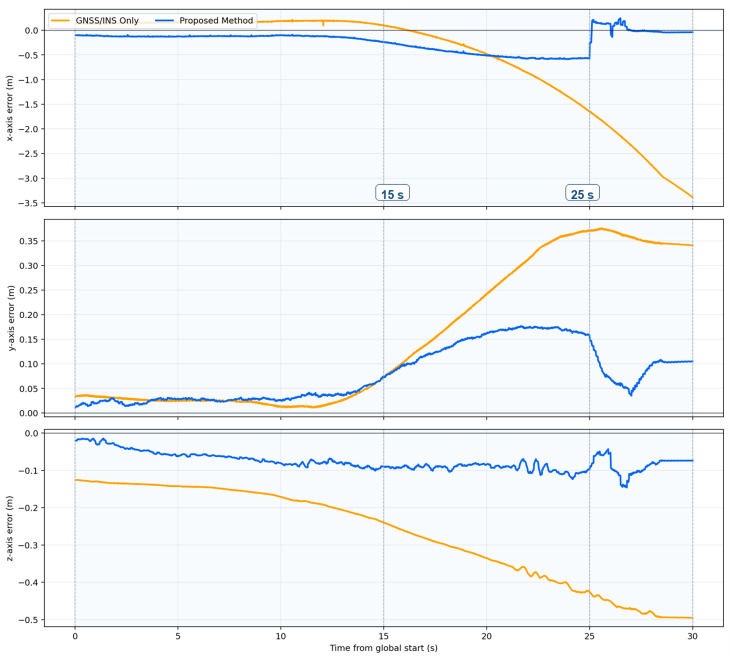
Axis-wise position-error time series. The dashed vertical guides indicate 15 s, when the GNSS/INS-only error begins to increase after tunnel entry, and 25 s, when the transition toward dedicated-sign-based correction begins.

Figure 11 compares the x-, y-, and z-axis errors over the 0–30 s time axis. The gap between GNSS/INS only and the proposed method widens toward the latter half of the sequence. The contrast is especially clear on the x- and y-axes, which correspond to the longitudinal and lateral directions of travel. After 25 s, the baseline error rises rapidly as drift accumulates inside the tunnel. By contrast, after recognition of the dedicated localization support sign, the proposed method keeps the interval-average absolute errors of all three axes below 0.1 m in the dedicated-facility correction interval. This behavior indicates that dedicated-sign-based correction suppresses both the dominant longitudinal drift and the lane-relevant lateral deviation.

**Figure 12:**
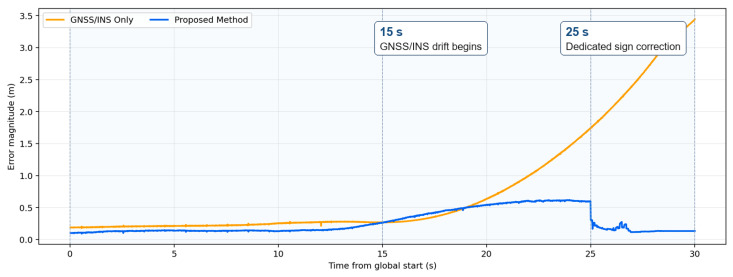
Overall position-error magnitude time series. The dashed vertical guides indicate the tunnel-entry-related error growth interval and the transition toward dedicated-sign-based correction.

The overall time series in Figure 12 shows the same trend. In the initial interval, both methods exhibit low error levels. The gap begins to widen after 15 s and expands markedly after 25 s. The abrupt change in the proposed-method curve is therefore not interpreted as instability of the LiDAR detector itself. Rather, it reflects a change in the dominant correction source within the fusion pipeline: before dedicated-sign recognition, the corrected output is influenced by ordinary mapped tunnel structures, whereas after recognition it is dominated by the dedicated localization support sign and its predefined map reference.

### 5.2. Analysis Results by Interval

**Figure 13:**
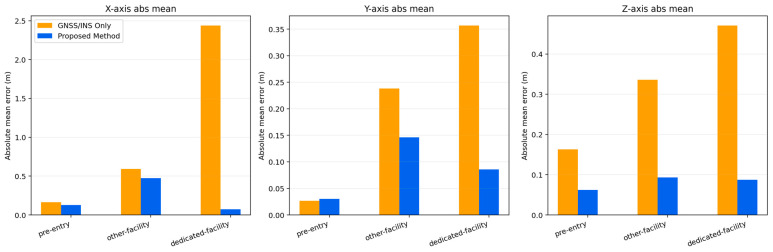
Interval-based axis-wise absolute-mean error comparison.

**Figure 14:**
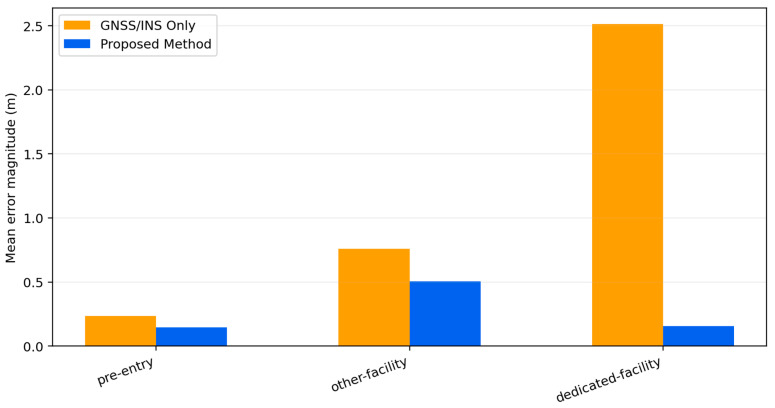
Interval-based overall mean error comparison.

In the pre-entry interval, the overall mean is 0.233 m for GNSS/INS only and 0.146 m for the proposed method. Both values are relatively small, indicating that GNSS/INS alone provides sufficient localization performance before tunnel entry. In the other-facility correction interval, the overall means increase to 0.759 m and 0.505 m, respectively. This shows that the proposed method still reduces error when only general tunnel structures are used, but the effect remains limited. The difference becomes much larger in the dedicated-facility correction interval after recognition of the dedicated localization support sign. In that interval, the overall means are 2.512 m for GNSS/INS only and 0.156 m for the proposed method. The |x| mean is 2.439 m for GNSS/INS only and 0.074 m for the proposed method. These results show that the proposed method is particularly effective for suppressing accumulated longitudinal drift. Figure 13 and Figure 14 provide an intuitive comparison of the interval-wise average errors, see Table 2.

This difference is operationally meaningful even when it appears to be only a few tenths of a meter. At the lane level, lateral error of several decimeters directly reduces lane-centering margin and can bias lane assignment near lane markings or tunnel-side boundaries. Longitudinal error of the same order shifts the timing of map-matched events and the estimated spacing to leading or following vehicles. The interval comparison therefore captures a practically important difference in path-following stability rather than a merely cosmetic numerical improvement.

#### 5.2.1. Comparison Between Other-Facility and Dedicated-Facility Correction

A key point of this study is that the dedicated-facility correction interval must be compared not only with GNSS/INS only but also with the immediately preceding other-facility correction interval. If correction based on general tunnel structures were already sufficient, there would be little need to design a dedicated localization support sign. This section therefore compares correction based on general tunnel structures with correction based on the dedicated sign. To standardize the comparison window, only the 20–25 s segment of the other-facility correction interval is used here.

This comparison is an internal ablation within the same registration pipeline, not a benchmark against State-of-the-Art tunnel-localization or LiDAR-inertial-odometry methods. Its purpose is to isolate the effect of replacing ordinary tunnel structures with a purpose-built LiDAR localization sign under the same vehicle, tunnel, and registration conditions.

In the 20–25 s other-facility correction interval, the overall mean of the proposed method is 0.592 m. In the 25–30 s dedicated-facility correction interval, it is 0.156 m. This corresponds to an additional error reduction of approximately 73.6%. The difference is even clearer on the x-axis. The |x| mean of the proposed method decreases from 0.559 m in the other-facility correction interval to 0.074 m in the dedicated-facility correction interval, which corresponds to an error reduction of approximately 86.8%. The |y| mean also decreases from 0.169 m to 0.086 m. These results indicate that correction based on the dedicated localization support sign is not merely a repetition of registration with tunnel structures. It provides a reference structure that is more stable and more accurate than ordinary tunnel structures, see Table 3.

#### 5.2.2. Dedicated-Facility Correction Interval

**Figure 15:**
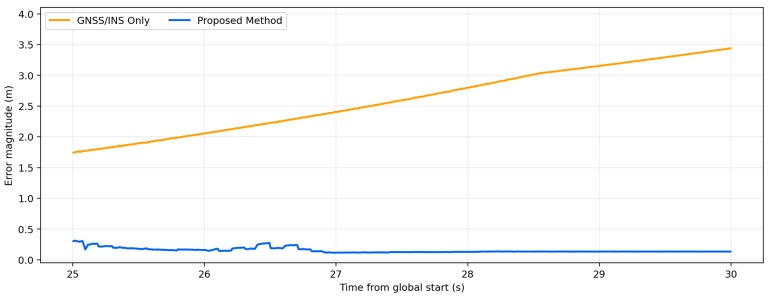
Focused overall position-error comparison in the 25–30 s dedicated-sign correction window.

**Figure 16:**
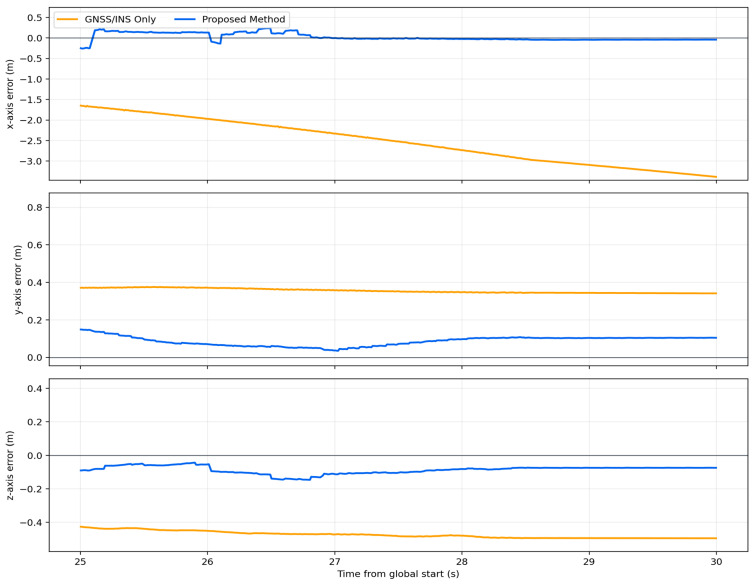
Focused axis-wise comparison in the 25–30 s window. Axis-wise errors are shown separately because longitudinal, lateral, and vertical deviations have different operational meanings in the vehicle coordinate frame.

The dedicated-facility correction interval is the section in which the effectiveness of the proposed correction pipeline is most clearly observed. In this interval, the proposed method reduces the |x|, |y|, |z|, and overall mean errors by approximately 97.0%, 75.9%, 81.5%, and 93.8%, respectively. Figure 15 and Figure 16 show the time-series behavior in this interval only. Figure 15 isolates the interval after dedicated-sign recognition and highlights the short-term convergence behavior of the corrected localization output.

After the dedicated localization support sign is first detected at around 25 s, short transient fluctuations remain for a brief period. As the vehicle approaches the sign, however, the results suggest that the point clouds become sufficiently stable from about 27 s onward, after which the errors converge. In particular, the x-axis error approaches a near-zero level within the analyzed window. This result supports the short-term effectiveness of the proposed correction pipeline after dedicated-sign recognition and suggests the potential for maintaining localization stability in longer shadowed sections if dedicated signs are installed at appropriate intervals and further validation is conducted.

**Figure 17:**
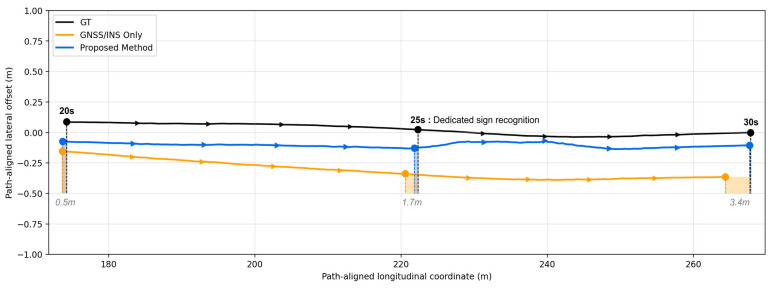
Path-aligned top-view comparison in the dedicated-sign correction section. The top-view representation emphasizes the lane-relevant lateral separation from the post-processed reference trajectory.

Figure 17 complements the time-series plots with a path-aligned top-view representation. The horizontal axis follows the driven path in the longitudinal direction, whereas the vertical axis shows the lateral offset from the ground-truth trajectory. After recognition of the dedicated sign near 25 s, the proposed method follows the ground-truth path closely and maintains the lateral offset within about 0.1 m, while GNSS/INS only continues to separate from the reference path as longitudinal drift accumulates. The widening gap toward 30 s shows that the late-stage difference is systematic rather than an isolated pointwise fluctuation.

## 6. Discussion

This study presented and validated a LiDAR-based localization-correction pipeline that combines a dedicated localization support sign with a point-cloud-registration-based correction algorithm. The goal was to mitigate localization drift in autonomous vehicles in GNSS-degraded tunnel sections. Across the three analysis intervals, the proposed method consistently produced lower mean position errors than the GNSS/INS-only baseline. The largest reduction occurred in the dedicated-facility correction interval, where the dedicated sign provided the most direct correction reference.

The proposed method also outperformed correction based on other tunnel structures. The overall mean error in the dedicated-facility correction interval was approximately 3.24 times smaller than that in the other-facility correction interval. In addition, the |x| mean, representing longitudinal drift along the travel direction, decreased from 2.439 m to 0.074 m, and the |y| mean, representing lateral deviation, decreased from 0.357 m to 0.086 m. Even differences on the order of a few tenths of a meter matter at the lane level: longitudinal bias affects map-matched event timing and vehicle-spacing estimates, whereas lateral bias directly reduces lane-centering margin. These results therefore suggest practical benefits for path-following stability, beyond a numerical reduction in position error.

The results further suggest that the dedicated localization support sign matters not only because it is easy to detect, but also because it provides a clearly defined support point, consistent geometry, and controlled installation conditions. Together, these characteristics enable more stable correction and smaller errors than those obtained from correction based on ordinary tunnel structures. At the same time, the study shows that a localization-correction strategy can be built using LiDAR alone under degraded conditions, which is valuable when auxiliary sensors become unreliable or unavailable.

The contribution of this study is not intended to be a new generic point-cloud registration theory. Rather, it lies in coupling a LiDAR-specific dedicated infrastructure reference with an online correction pipeline so that the registration problem becomes more repeatable in GNSS-degraded tunnel sections.

From a practical deployment perspective, the proposed sign is not intended to be installed uniformly throughout every tunnel section. Rather, it can be selectively deployed at GNSS-vulnerable locations where localization drift is likely to accumulate, such as long tunnels, tunnel entrances and exits, curved tunnel sections, and underground roads. Because the sign serves as a sparse correction anchor, its installation spacing can be determined according to the expected drift accumulation, LiDAR observation range, vehicle speed, and road geometry. For real-world deployment, however, standard specifications for installation spacing, mounting height, reflectivity, contamination control, and damage inspection should be established.

Several limitations should be noted. The validation was conducted in a nationally certified proving-ground tunnel that reproduces real-road conditions, but the tested environment does not fully represent the diversity of real tunnel operations. Therefore, the results should be interpreted as controlled proving-ground evidence that a dedicated LiDAR-observable sign can improve localization correction under the tested tunnel geometry, rather than as proof that the same numerical accuracy can be achieved in all tunnel environments. Because of safety constraints and the need for repeatable experiments, the same validation has not yet been carried out in real traffic. The results may vary depending on tunnel length, curvature, cross-sectional shape, traffic density, vehicle speed, LiDAR mounting configuration, and the spacing and installation condition of the dedicated signs. At higher speeds, the available observation time for the dedicated sign becomes shorter, and the number of LiDAR frames containing the sign may decrease, which can affect detection confidence, point-cloud density, and registration stability. In addition, the 5 s dedicated-facility correction interval demonstrates short-term correction behavior after sign recognition, but it does not prove long-term stability over extended tunnel sections. Moreover, this study did not comprehensively address conditions such as occlusion by preceding or large vehicles, illumination changes, rain, fog, contamination, or sign damage. Therefore, the robustness of the proposed pipeline under adverse weather, severe occlusion, sensor malfunction, contamination, and physical damage to the sign remains to be verified.

Regarding map accuracy, the reference map was generated through post-processed GNSS/INS and LiDAR-based mapping. While it served as a reliable baseline for this study, incorporating independently surveyed control points or a survey-grade reference map in future validation would allow for an explicit quantification of absolute map uncertainty. Furthermore, this study focused primarily on evaluating the efficacy of a LiDAR-observable external infrastructure reference within a standard GNSS/INS and LiDAR-registration pipeline. Consequently, integrating wheel-speed/steering-angle sensors or recent LiDAR-inertial odometry (LIO) baselines was outside the current scope. A direct performance comparison with these odometry-fusion and LIO methods under identical tunnel conditions remains an objective for future research.

Future studies should therefore verify the recognition and correction performance of the dedicated localization support sign in real traffic environments. They should also evaluate robustness under partial occlusion, adverse weather, and nighttime conditions. Further validation is needed in a wider range of GNSS-vulnerable environments, including urban canyons, underpasses, and deep underground roads. Deployment strategies and maintenance standards should also be established so that dedicated-sign-based correction can be applied continuously over longer road sections.

## 7. Conclusions

This study proposed and validated a LiDAR-based localization-correction pipeline using a dedicated localization support sign for GNSS-degraded tunnel sections. The proposed method integrates sign detection, point-cloud registration, and pose correction into a single workflow so that the dedicated sign can serve as a repeatable geometric reference for localization correction.

The experimental results showed that the dedicated localization support sign reduced localization drift more effectively than GNSS/INS-only localization and correction based on ordinary tunnel structures within the same registration pipeline. In particular, the proposed method substantially reduced the overall mean position error after the dedicated sign was recognized, demonstrating the value of purpose-built LiDAR-observable infrastructure as a sparse correction anchor. Overall, this study shows that the dedicated localization support sign is not merely a visibility aid but can function as sparse, high-value localization infrastructure with practical potential for future roadway deployment.

However, the validation was conducted in a controlled proving-ground tunnel at a limited driving speed and over a short correction interval; therefore, further evaluation is required before the results can be generalized to real traffic conditions, highway-speed driving, longer tunnel sections, adverse weather, partial occlusion, and different tunnel geometries. Future work should also compare the proposed framework with GNSS/INS/vehicle-odometry fusion methods, recent LiDAR-inertial odometry frameworks, and tunnel localization methods, and establish deployment and maintenance standards for practical roadway application.

## Figures and Tables

**Table 1:** Definition of analysis sections.

Section	Time Range	Analysis
Pre-entry	0–15 s	Before entering the tunnel and near the tunnel boundary
Other-facility correction	15–25 s	Stage after entering the tunnel but before recognizing the dedicated sign; section where the correction pipeline based on other tunnel structures within the tunnel is active
Dedicated-facility correction	25–30 s	The section where the dedicated correction pipeline operates after recognizing localization support sign

**Table 2:** Interval-wise position errors of GNSS/INS only and the proposed method.

Method	Interval	$\left|x\right|$ Mean (m)	$\left|y\right|$ Mean (m)	$\left|z\right|$ Mean (m)	Overall Mean (m)
GNSS/INS Only	Pre-entry (0–15 s)	0.163	**0.027**	0.163	0.233
**Proposed Method**		**0.127**	0.030	**0.062**	**0.146**
GNSS/INS Only	Other facility correction (15–25 s)	0.593	0.238	0.336	0.759
**Proposed Method**		**0.474**	**0.146**	**0.093**	**0.505**
GNSS/INS Only	Dedicated-facility correction (25–30 s)	2.439	0.357	0.471	2.512
**Proposed Method**		**0.074**	**0.086**	**0.087**	**0.156**

Note: Bold values indicate the lower error value between the compared methods within each interval.

**Table 3:** Position errors in the comparison window for other- and dedicated-facility correction.

Method & Interval	$\left|x\right|$ Mean (m)	$\left|y\right|$ Mean (m)	$\left|z\right|$	Overall Mean (m)
Proposed Method & Other-facility correction (5 s)	0.559	0.169	0.096	0.592
GNSS/INS Only & Dedicated-facility correction	2.439	0.357	0.471	2.512
**Proposed Method & Dedicated-facility correction**	**0.074**	**0.086**	**0.087**	**0.156**

Note: Bold values indicate the lower error value among the compared rows.

## Data Availability

The algorithms and codes utilized in this study are available at the following GitHub repository: https://github.com/RideFlux/rf_roadsign_detector (accessed on 17 June 2026).

## Abbreviations

The following abbreviations are used in this manuscript:

ADS	Autonomous Driving System
BEV	Bird’s-Eye View
CNN	Convolutional Neural Network
GNSS	Global Navigation Satellite System
HD map	High-Definition Map
ICP	Iterative Closest Point
INS	Inertial Navigation System
KD-tree	k-dimensional tree
PCA	Principal Component Analysis
PFN	Pillar Feature Net
PG	Proving Ground
SLAM	Simultaneous Localization and Mapping
VFE	Voxel Feature Encoding

## Appendix A. Key Terms Used in This Study

The following key terms are used in this manuscript:

Dedicated support sign	Purpose-built roadside reference for LiDAR-based localization correction.
Localization-support point	Predefined corner point linking the detected sign to mapped absolute coordinates.
Correction pipeline	End-to-end workflow of sign detection, source–target registration, and pose update.
Other-facility correction	Correction from ordinary tunnel structures before dedicated-sign recognition.
Dedicated-facility correction	Correction after dedicated-sign recognition in the late interval.
